# Local knowledge of traditional fishermen on economically important crabs (Decapoda: Brachyura) in the city of Conde, Bahia State, Northeastern Brazil

**DOI:** 10.1186/1746-4269-8-13

**Published:** 2012-07-02

**Authors:** Henrique Fernandes de Magalhães, Eraldo Medeiros Costa Neto, Alexandre Schiavetti

**Affiliations:** 1 Biologist, Master of Science in Applied Zoology, Universidade Estadual de Santa Cruz, Ilhéus, Brazil; 2 Biologist, Master of Science in Development and Environment, UFAL, Brazil. PhD in Ecology and Natural Resources, UFSCar, Brazil. Post-doctoral studies in Ethnoentomology, UNAM, Mexico. Permanent Professor at Universidade Estadual de Feira de Santana, Feira de Santana, Brazil; 3 Ecologist. Master of Science in Environment Engineering Sciences, USP, Brazil. PhD in Ecology and Natural Resources, UFSCar, Brazil. Full Professor at Universidade Estadual de Santa Cruz, Ilhéus, Brazil

**Keywords:** Artisanal fishing, Crustaceans, Mangroves, Ethno-ecology

## Abstract

**Background:**

This article records the traditional knowledge of crab gatherers in the city of Conde, in the North Coast Region of Bahia State, Northeastern Brazil.

**Methods:**

Data on biological and ecological aspects of economically important brachyuran crustaceans have been obtained from semi-structured interviews and *in loco* observations conducted from September 2007 to December 2009. A total of 57 fishermen of both genders, aged between 10 and 78 years have been interviewed (individually or collectively) in different contexts; interviewees were asked about aspects such as external morphology, life cycle, trophic ecology, and spatial and temporal distribution of the major economically important brachyuran crustaceans in the region. Seven fishing communities were visited: Siribinha, Sítio do Conde, Poças, Ilha das Ostras, Cobó, Buri and Sempre Viva. Data were analyzed by comparing the information provided by participants with those from the specialized academic literature.

**Results:**

The results show that artisanal fishermen have a wide ranging and well-grounded knowledge on the ecological and biological aspects of crustaceans. Crab gatherers of Conde know about growth and reproductive behavior of the animals they interact with, especially with regard to the three major biological aspects: “molt”, “walking dance” and “spawning”.

**Conclusion:**

This knowledge constitutes an important source of information that should be considered in studies of management and sustainable use of fishery resources in the North Coast Region of Bahia State.

## Background

Mangrove ecosystems are typical of tropical and subtropical regions, in areas influenced by tidal movements [1]. They comprise ecosystemic units with specialized function as they are salinized environment, given the constant inundation by sea water due to the tidal movement. These ecosystems are considered of fundamental ecological importance in their areas of occurrence [2], and as such mangroves should be categorically classified as permanent protected areas since they maintain fish production at adjacent regions and ensure the stabilization of coastal formations. Additionally, two-thirds of the world’s fishing communities depend on their existence [3-5].

Crustaceans play a role in the ecosystem dynamics not only by their function in the food chain, but also because some of them – the decapod brachyurans – constantly modify the substrate as they dig holes and bring organic matter from the lower stratum to the surface [6]. Brachyurans are a diverse group and one of the largest biomasses in marine and estuarine environment [7]. These crustaceans are fishery resources of high prestige for human communities inhabiting estuarine zones and the exploitation of these resources provides a livelihood for many of them [8-10]. This group of animals is culturally used for various purposes: handicraft [11,12], as a source of income and protein [13-15], in folk medicine [16-18] and in playful activities [11]. Cultural interactions between humans and crustaceans are studied by ethnozoology that, paraphrasing Posey [19], can be defined as the field of ethnobiology that investigates knowledge, classification and methods of use of animals by human societies.

In Bahia State, Northeastern Brazil, fishing activities are especially characterized by familiar work, where all members of the family are directly involved with the collecting and processing of those resources [20]. Specifically in the local mangroves, brachyuran crustaceans are amongst the main wildlife resources that are found and extracted, such as *Ucides cordatus* (Linnaeus, 1763), *Cardisoma guanhumi* (Latreille 1825), *Goniopsis cruentata* (Latreille 1802), and swimming crabs of the family Portunidae. In addition to serving as food, some species are also used for medicine, as craft, and leisure (18).

Considering ethnozoological studies related to crustaceans, there are few works carried out in Bahia State. Costa-Neto [21] has discussed about the ethnobiology and ethnotaxonomy in fishing communities in the municipality of Conde, however fish were the main surveyed animal group; Costa-Neto and Lima (18) studied the local uses of mangrove crustaceans by the inhabitantys of Siribinha beach; Souto [22] carried out an ethnoecological approach of the collecting of *Ucides cordatus* in Acupe beach; Saraiva [23] recorded some ethnoecological aspects of *Macrobrachium carcinus* (Linnaeus, 1758) in the city of Camaçari; Magalhães [24] investigated about some brachyurans of economic importance to artisanal fishing communities in the city of Conde.

In the last decades, the northern coast of Bahia State has faced profound political, socio-economic and cultural changes due to their insertion in the global development model, especially due to the expansion of national and international tourism in the region [20]. Consequently, the local landscape and biota have been undergoing changes that have aggravated the existing pressures on ecosystems, thus subjecting the traditional populations to activities with several degrees of impacts generated by the tourism industry [20]. The fishing activity has been gradually replaced by tourism related jobs, what means a progressive loss of knowledge about fishing gear and sustainable management techniques of fishery resources through the generations; that has also negatively reflected on the local fauna and flora [25].

Given the progressive impacts the North Coast region has been suffering over the last decades and considering its socio-cultural and ecological relevance, this study broaches the local knowledge of artisanal fishermen communities in the municipality of Conde by recording biological, ecological and behavioral aspects of economically important brachyuran crustaceans.

## Methodology

**Figure 1:**
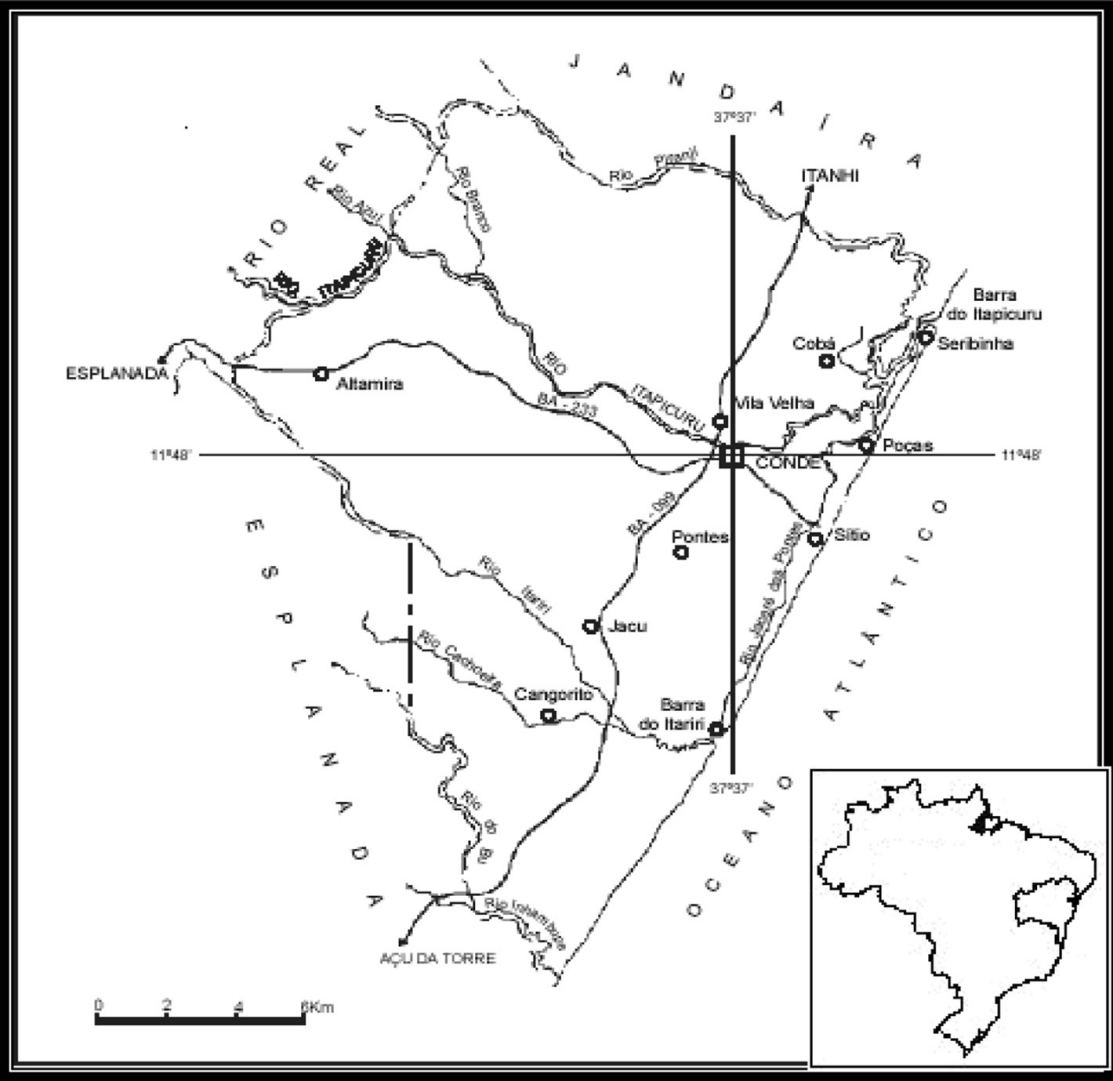
**Location of the city of Conde and some of the major fishing communities studied. Adapted from Costa-Neto**[18]**.**

As shown in Figure 1, the study area is included in the “Área de Proteção Ambiental Litoral Norte” (Environmental Protection Area of the North Coast), in the city of Conde, more precisely in the estuarine and coastal area of the lower course of Itapicuru River. In its initial stages, the fieldwork was conducted between September 2007 and October 2008, when monthly visits were made to the communities of Siribinha, Poças, Sítio do Conde, Ilha das Ostras, Cobó, Buri and Sempre Viva. In the second stage of the research, two fortnightly visits were made to the three last communities, in the months of February, May and December 2009. When selecting the visitation months, it was sought to consider the two seasons of the year recognized by the fishing communities: “summer” (September-March) and “winter” (April-August), thus correlating the data obtained during the seasons.

Data were recorded by means of semi-structured interviews and through behavioral observations, using the usual ethnographic data collection techniques, following ethnoscientific notions with a balance between emic and etic focused approaches [26]; on the other hand, observations had an ad libitum feature. The questionnaire was approved by the Research Ethics Committee of the State University of Feira de Santana (Comitê de Ética em Pesquisa da Universidade Estadual de Feira de Santana: CEP-UEFS) based on Resolution no. 196/1996 of the National Council of Health, which governs the ethical aspects of research involving humans. A free and informed consent form was read out and made available to those who participated in the study. The aims of the research were explained clearly at the beginning of each interview and those involved were asked whether they would like to participate in the study. The interviews (whether individual or collective) occurred in various contexts, with the participation of 48 men (84.2%) and 9 women (15.8%) whose ages ranged between 10 and 78. The respondents were fishermen of both genders, specialists in gathering mangrove crabs *Goniopsis cruentata*, swimming crabs (Portunidae), and land crabs *Ucides cordatus* and *Cardisoma guanhumi*. They were contacted according to the snowball technique [27], in which a local specialist indicates another one and so on. Women use to go to mangrove forest in order to collect “aratu” *G. cruentata* and swimming crabs popularly known as “siris”, while male fishermen collect land crabs and some (16 of them) are specialized on collecting species of Portunidae. The interviews were recorded using digital recorders and later transcribed for analysis.

Body topography has also been recorded; to this end, cards describing schematic drawings of a land crab [28] and a swimming crab [29] were distributed to the interviewees in order to register the local names they give to the external body structures.

Aiming to observe and collect brachyuran specimens, excursions guided by locals were made to the mangrove. The technique employed during these field trips was the tour [30], i.e., when the researcher follows a path determined by the research participant, which in turn describes what happens. These collections allowed conducting projective tests, which consisted of the presentation of both photographs and visualizations in the natural places of crustacean species, so that the respondents described their perceptions on the biology and ecology of crustaceans.

The data were analyzed using the union model [31]. According to this model, all available information on the surveyed subject is to be considered. Local information provided by the participants was compared with those from the specialized academic literature. Based on synchronic and diachronic interviews, the controls were performed through verification tests of consistency and validity of responses [31]. All ethnographic material (recordings, transcriptions, field notes and photographs) is stored at the Laboratory of Ethnobiology and Ethnoecology of Universidade Estadual de Feira de Santana, to the attention of the curator of the Ethnozoology Section.

The specimens collected were processed and identified by Professor M. Sc. Cesar Carqueija (Faculdade de Tecnologia e Ciências in Salvador) to the lowest possible taxonomic level, and afterwards stored in the Zoology Museum of the FTC. The record numbers are: *Ucides cordatus* (MZFTC 5879), *Cardisoma guanhumi* (MZFTC 5880), *Goniopsis cruentata* (MZFTC 5881), *Callinectes exasperatus* (MZFTC 5882), and *Callinectes danae* (MZFTC 5883). Duplicates were stored in the invertebrate collection of the Zoology Museum of Universidade Estadual de Feira de Santana (MZUEFS) and also in the zoological collection of the Universidade Estadual de Santa Cruz.

## Results and Discussion

### General knowledge about the morphology of brachyuran crustaceans

In the cognitive system of the artisanal fishermen of Conde, were identified three general patterns used in the construction of body topography. They are: polyonomy, i.e., the use of more than one designation to the same body structure; the functionality assigned, which designates functions to the respective structures named; and the anthropomorphic analogy, hence demonstrating the influence of terms of human morphology in the naming of body parts of brachyuran. These same patterns were recorded by Souto [15] during the elaboration of the topography of crustaceans, fishes and mollusks, in a study conducted at the fishing community of Acupe, Bahia.

The external body structures of crustaceans receive designations that reflect how locals perceive these animals. For example, the terms “beard”, “arm” and “mouth” are used to designate antennas, chelipeds and chelae, respectively. Fishermen designate specific functions to the structures of the body of brachyuran crustaceans (Table 1). The first pair of pereopods (chelipeds), called “arms” or “spikes”, and the chelae, called “mouth” are meant to “get the food”. Souto [15] probably attributed the origin of the word “mouth” to a legacy of the early settlers, since the term was already used by Gabriel Soares de Souza in 1587 to designate such structures [32]. In the city of Conde, the term “mouth” was also used to designate the oral cavity of animals, whose function was also related to feeding; probably for that reason, the terms were associated. The remaining pairs of pereopods, called “hams”, are used for locomotion (“for walking”). On the other hand, the last pair of legs in swimming crabs (Portunidae) is known as “paddle”, in allusion to the structure responsible for the transportation of canoes in the aquatic environment.

**Table 1:** Cognition compared between indigenous and scientific knowledge related to the body topography of economically important brachyurans in the city of Conde

Local nomenclature	Scientific nomenclature	Function designated (emic conception)	Function attributed (etic conception)
Hull	Carapace	Protect	Protection
Beard	Antennas	Orient	Sensorial
Eye	Eye	See	Sight
Mouth	Mouth	Eat	Food ingestion
Chest	Thoracic sternums	Protect what is inside	Exoskeleton
Cover	Abdomen	Distinguish males and females	Protection of sexual organs
Legs	Pereopods	Walking	Locomotion
Joints	Carpus	. . .	Joint
Nail	Dactyl	Holding on the ground	Support on the substrate
Arm (gaff)	Cheliped	Defense	Defense
Mouth	Chela	Hold food and put it into the mouth	Get food
Oar	Pereopod	Swimming	Swimming

Sources: Ruppert et al. [7] and Brusca and Brusca [33].

Some body structures are used in sexual differentiation, especially the abdomen (“cover”) and the chelipeds (“arms” or “spikes”): the male has a narrower abdomen and different sized chelipeds, whereas the female has a longer abdomen and equal sized chelipeds. Body size of crustaceans was also indicated as a criterion for determining sexual dimorphism; in that, males would tend to grow larger than females. Sexual dimorphism observed for land crab (*Ucides cordatus*) is also determined by the presence of hair in the appendices of males, as noted by these two informants: “The difference is that males have hair on their hands” (Mr. F., 41 years old);“[…]the fingers of males are hairy, different from the females” (R., 29 years). Pinheiro and Fiscarelli [34] and Souto [15] have also reported the use of hair as an indicator of sexual differentiation in *U. cordatus*.

Another criterion used by the fishermen of Conde to distinguish the gender of crabs was the type of track the animals made, which could be either footprints in the mud or feces deposited, whose size and shape allowed determining the gender; the following statements show it clearly:

"“As the female is not hairy, its track is narrower than the male’s, which is wider” (Mr. N., 44 years old);"

"“[…] I myself can differentiate them even by their feces. It’s because the feces of females are tiny and thick. So, if the feces are like that, it is a female. If it is a long and thin, it is a male” (Mr. N.L., 32 years old)."

**Figure 2:**
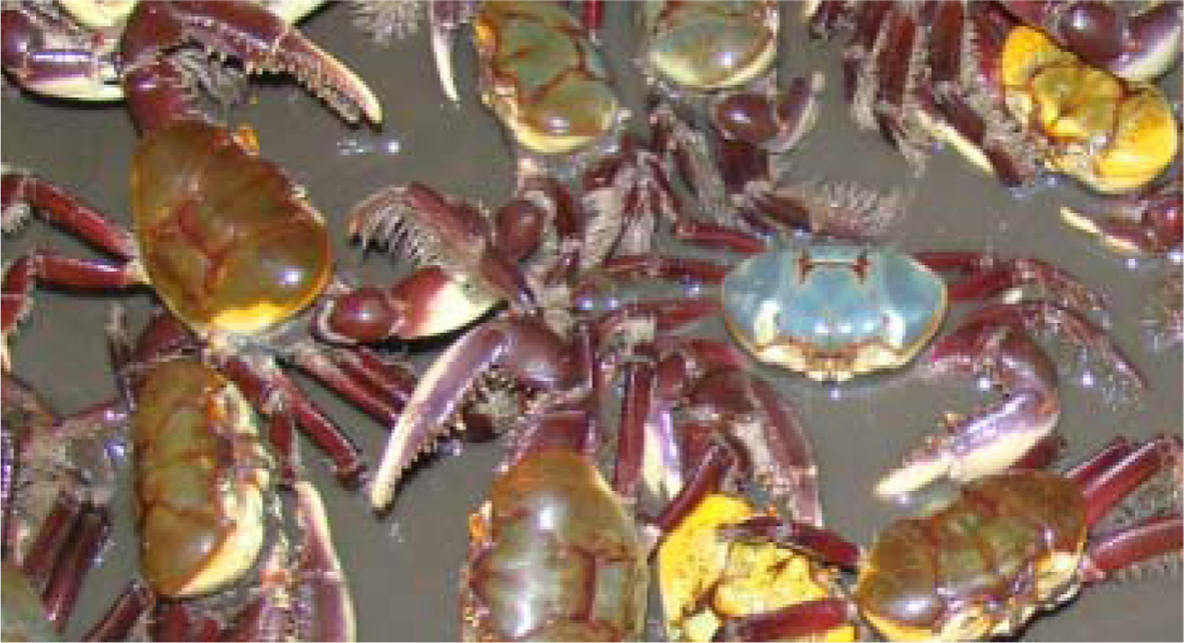
**Different color patterns for*Ucides cordatus*in the fishing community of Sempre Viva, Conde, Bahia State.** Photo by Henrique F. de Magalhães.

There may be morphological differences between specimens of the same species, especially with respect to body color (Figure 2). According to the reports of some people, the major factor that changes the color of *U. cordatus* and *Cardisoma guanhumi* is the physiognomy of the mangrove where the animal lives; that is confirmed by the following reports: “Much depends on the part of the mangrove where it lives, because there is a drier area in the mangrove. There’s a drier area and the crabs living there have another color. Those living in moister areas have different colors” (Mr. N.L., 32 years old). This information is correlated with the concept of Pinto-Coelho [35], which implies that “an ecological unit is the gathering of all organisms, which in turn interacts with the physical environment and is exposed to its influences.”

### Life Cycle

Crab gatherers of Conde know about growth and reproductive behavior of the animals they interact with, especially with regard to the three major biological aspects: “molt”, “walking dance” and “spawning”. Crab ecdysis (“molt”) occurs between June and September, a period that, according to local reports, includes winter and early summer. The beginning and the end of the changes directly depends on the tidal movement and the alternation of the lunar cycles; both stages occur during the “low tide”, when the mangrove substrate is dry, although it is still wet due to the previous tide. This notion was recorded by Alves and Nishida [5] as it was used by crab gatherers in a mangrove area located at the estuary of the Mamanguape River (Paraíba State). In the same study, the authors have found a significant reduction in the number of “puddles” during “low tide”, which they believed to have been due to the fact that the mud was already very hard in that period, thus hindering the excavation of the galleries (“burrows”) by crabs. According to the following reports, this finding was also verified in Conde:

"“[…] this time of the year they are all puddled because that is to change the hull […]. It's all puddled. You can go through and see their holes all over there. Then, nobody can catch them at that time. Only in October and November” (Mr. F., 41 years old)."

""During the neap tides we get them more often. During neap tides it is better, because the soil is dry, the mud is hard and they cannot dig as much, right? And when the tide is high it becomes harder, because it seems like they sink deeper; as the ground is softer, they sink” (Mr. N., 44 years old)."

It is during ecdysis that crabs burrow into the substrate and undergo carapace change. Ecdysis, or “molt”, is necessary for crabs to grow, since their exoskeleton is not alive and do not grow with the animal [36]. This phenomenon usually occurs once a year, in adults, and is more commonly observed in young crabs [5], especially when they burrow into individual galleries, under the mangrove trees, at an approximate depth of one meter [37]. Reproduction, behavior, and metabolic processes of many crustaceans are directly affected by the physiology of molt cycle [36]. At that stage, the animals become more fragile and vulnerable to natural predators [38,39]. According to Maneschy [40], the crabs become lean and little tasty soon after the change in carapace. This morphological variation in the body of crabs may be evidenced in the following testimonial provided by an artisanal fisherman from the Cobó community: “They are now so short, but when they leave, they are large. So fat, but when it leaves, it is thin. They totally change their bodies” (Mr. F., 41 years old).

The periods of “walking dance” (mating) and spawning of *U. cordatus* and *C. guanhumi* usually cover the months from December to March (summer) and occur when the moon is full and the tide starts to “lower”. That stage affects the reproductive behavior of these animals, which become disturbed (“Due to the walk, they get ‘insane’ when it is a flooded period”, Mrs. E., 72 years old). This phenomenon is not observed for “aratu” (*Goniopsis cruentata*) and swimming crabs (Portunidae), which, according to local reports are “wanderers by nature”, despite spawning occurs in the same period. The following reports exemplify the local knowledge about the “walking dance” behavior:

""In January, the walking dance occurs during high tide. When it drains, they do not leave, because they are reproducing” (L., 13 years)."

"“He [referring to the aratu crab] is a wanderer by nature, right? They scuttle back and forth. It is not like the other crabs, which have holes. They are constantly walking” (Mrs. L., 34 years old)."

""Ii begins in December. From December to March, four walking dances occur when the moon is full” (Mr. E.V., 75 years old)."

"“When the tide comes at 2 a.m. and it falls three times, it’s when they start to walk. No matter if it is day-time, but they prefer the night-time" (Mr. E., 75 years old)."

“Walking dance” is a designation used by coastal communities by referring to the behavior of male and female *U. cordatus* as they leave their burrows and walk on the mangrove sediments [41]; this is considered the major reproductive event of the species [42]. Góes et al*.*[41] have observed that the main reason for this phenomenon is the fact that females need to climb the roots of mangrove trees for egg extrusion (spawning). In other regions, this event is known as “carnaval do caranguejo” (“carnival of the crabs”) [38].

According to Sastry [43], crustaceans can reproduce during all months of the year (continuous pattern) or only in the months when environmental conditions are more favorable (discontinuous patterns or seasonal). Brachyurans display an impressive array of reproductive patterns that may be related to environmental factors [44]. Studies by Alcantara-Filho [38] and Costa [45] have described that the reproductive period of *Ucides cordatus* occurs from December to May. These authors have found that after reproduction in the summer, this crab undergoes ecdysis, with a peak in autumn; on the other hand, females with gonads in maturation process were only found in late winter. According to Hartnoll and Gould [44], this fact corroborates the assertion that growth and reproduction are antagonistic processes that compete for the same resources. Shorter periods have also been described: from January to May [46] and from January to March [47]. Dalabona and Silva [48] mention that studies on the reproductive period have biological importance, especially for commercially exploited species, thus allowing the elaboration of protection laws that contribute to the maintenance of population stocks.

### Trophic ecology

Knowledge of the artisanal fishermen interviewed on the trophic ecology of crabs involves two major aspects: diet composition and food chain they belong to. According to Marques [49], this knowledge is utilitarian, since a cumulative empirical knowledge about the resource/prey leads to a foraging/predating behavior. The following excerpts confirm that this assertion is applicable in Conde:

""I live from fishing. I only make money from fishing” (Mr. F., 41 years old)"

"“We live here from fishing, right?” (Mr. J., 46 years old)."

"“It is because fishing is our art, right? We know that because we live here” (R., 29 years old)."

According to crab gatherers from Conde, the “uçá” *U. cordatus* and the “gaiamum” *C. guanhumi* have a very restricted diet that largely consists of leaves and roots of trees that live in the mangrove. Analogue ethnoecological studies have reported similar data [15,22,50]. In the community of Acupe (Santo Amaro, Bahia), Souto [22] has also recorded the testimonials of local artisanal fishermen who reported that white mangrove propagules (*Laguncularia racemosa*) are part of the diet of *U. cordatus* crabs.

Zoological studies on the trophic ecology of crabs, particularly the land crab, indicate that these animals are omnivorous, instead of obligatory herbivorous [45,47,51,52]. By analyzing the stomach content of *U. cordatus* in a mangrove forest of Santa Catarina State, Branco [53] has found that 95% of food was of plant origin, but also some decaying organic matter of animal origin and minerals. This crab plays a role in the trophic web of mangroves, as it collaborates in the processing of the leaves that fall off trees by incorporating their nutrients into the soil and therefore increasing availability of food in the detritivore chain [50,53].

On the other hand, the diet of swimming crabs and *Goniopsis cruentata* is broader, since it includes vegetable matter (leaves and fruits of mangrove trees) and decomposing organic matter, including dead animals. According to Conde’s artisanal fishermen, these crustaceans are omnivorous; this knowledge is therefore consistent with information contained in the specialized scientific literature [51,52,54-57]. The following statements make such perception evident:

"“They (referring to the Portunidae family crabs) eat the things of the river, fish. They like something more than bad, rotten. Even shrimps may be eaten by them” (R., 29 years old)."

"“Aratu (eats) dead and rotten things there in the mangrove” (R., 29 years old)."

In Armação do Itapocoroy (Penha, Santa Catarina State), Branco and Lunardon-Branco [58] have recorded a diet consisting of 24 items of plant and animal origin for *Portunus spinimanus* Latreille, 1819 (Portunidae), where crustaceans and fishes were observed to be the most abundant resources. Gaspar [59] asserts that, since the swimming crabs are voracious hunters and insatiable carnivorous, they prefer feeding on decomposing organisms and can digest all organic material; this eating habit (saprozoic) that makes the Portunid crabs major elements in the promotion and maintenance of cleanliness on brackish water areas and beaches.

With respect to the role of “aratu” (*G. cruentata*) in the trophic web of mangroves, further bio-ecological studies are needed to strengthen this knowledge, including primary herbivory, predation, and exportation of biomass and energy [57].

Based on the information above, it is possible to observe an overlapping of ecological niches among brachyuran decapod crustaceans; such concept refers to the use of the same types of resources by two or more species [60]. Once brachyuran crustaceans share essentially the same area (sympatric), there is also competition among them [61].

As pointed out by artisanal fishermen in the city of Conde, some of the major predators of brachyuran crustaceans in the region are fishes, mammals and humans (“If I am not mistaken, man is the worst of them all”, Mr. C., 67 years old). Animals such as raccoons (*Procyon cancrivorus* Cuvier 1798), otters (*Lontra longicaudis* Olfers 1818), monkeys and the opossum (*Didelphis* sp.) are of great importance. These animals and humans can be considered direct competitors within the food chain, since they compete for resources available in the same environment; this can be verified in the following reports:

"“There are the raccoon, the opossum. But when the opossum goes in and it cannot exit, then it dies. Not the raccoon. The raccoon strips, puts its arm in and pulls it out” (Mr. E.V., 75 years old)."

"“This one, there is one we call it otter. So it goes there and drills a hole, right? Then, when we come here, the damage is done. But what can we do? It is the work of nature. They also need to eat, right?”(Mr. A.N., 71 years old)."

Nomura [62] has made a collection of reports from folklorists on crab-eating raccoons; Emmons and Feers [63] describe it as an inhabitant of mangroves, which feeds on mollusks, fishes and crabs. The presence of otters in different environments, including mangroves, is directly associated with food availability [64]; besides, their diet preferably consists of fishes, followed by crustaceans, birds, reptiles and small mammals [65].

### Spatial and temporal distribution

The knowledge of fishermen of Conde on the temporal and spatial distribution of brachyuran crustaceans with important role in local economy is closely related to other ecological knowledge regarding these animals, such as migratory flow, habitat types, life cycle, and foraging areas.

There are two major seasons as determinants in the distribution of crabs along mangroves and adjacent areas: “summer” and “winter”, which, as pointed out by Marques [31], do not correspond to the official seasons of the annual cycle, but to periods related to the rain and drought. In view of crab gatherers of Conde, summer occurs from September to March, while winter begins in late March and ends in August. In the study by Costa Neto [21], artisanal fishermen of Siribinha (Conde, Bahia) have reported a similar frequency, with summer occurring from mid-August and until the Holy Week period (between the second half of March and the first fortnight of April) and winter starting then and continuing until mid-August. Mourão and Nordi [65] have also found similar information from artisanal fishermen of Mamanguape River estuary (Paraíba), with summer between September and February, when the water begins to “clean up” and winter from March to August.

Fishermen of Conde directly associate the seasonality of brachyuran crustaceans gathered for commercial purposes with the local economic productivity. According to them, summer is generally the most productive season (“For us who live from fishing, summer is wonderful”), except for swimming crabs, which “are more common in the winter”, as mentioned in the following excerpts:

"“It is available from December to March. After the walking dance, in March, there is a tendency to reduce (Mr. F., 41 years old)."

"“Yeah, the swimming crabs can be caught the whole year, but I am quite sure it can be found in winter. In summer it reduces even a hundred percent. On the other hand, the aratu can always be found. But the aratu also reduces in winter because it disappears, right?” (Mr. F., 41 years old)."

"“The swimming crab can be found all year long, but this time of the year, in August there is a lot of if, but there is not much in summer. It keeps on available, but there is not much” (Mr. E., 75 years old)."

The definition and perception of these two major seasons are associated with the understanding of gatherers of Conde on the variations of water salinity in mangroves, which causes changes in the spatial distribution of species and consequently seasonal variations; the following testimonials corroborate that:

"“The land crab (referring to Ucides cordatus) can be more easily found in December. I think it is because of the time, right?” (Mr. E., 75 years old)."

"“The swimming crabs depend on the flooding. At that time, the river flow drags them to the sea. When the water starts to get salty, they all come back to the river” (Mr. R., 66 years old)."

According to Posey [66], the spatial distribution of crabs is expressed by artisanal fishermen of Conde as major ecological zones or “ecozones”. The author has defined “ecozones” as ecological areas where resources can be found and recognized in other cultural systems that may or may not coincide with those scientific typologies. The major ecological zones observed in Conde are locally known as “mangrove”, “river” and “marsh”. The land crab (*U. cordatus*), the blue crab (*Callinectes exasperatesCallinectes danae*) and the mangrove crab (*G. cruentata*) are the crustaceans of the “mangrove”; the other swimming crabs inhabit the “river”, whereas the “marsh” is the habitat of “gaiamum” crabs. Within a single “ecozone”, there are different areas of distribution of faunistic resources. Thus, for example, *U. cordatus* lives “holed up” (“under the mud”), in holes whose depths are approximately a hundred or a hundred and twenty centimeters, according to the locals (“It is approximately one hundred and twenty centimeters deep”, Mr. J., 46 years old); *G. cruentata* lives in “stock hollows” or “mangrove swamps”, although it may occasionally invade the burrows of *U. cordatus* when the tides are “neap”; in turn, the blue crab inhabits the banks of the rivers that flow through the mangrove, although it can be also found “holed up”. Yet, these spatial distributions may change occasionally; as an example, the “gaiamum” crabs may leave their burrows in the “marshes” and move to the mangroves (according to local reports, it is rare though) when the “ground is hard”. The following testimonials corroborate this information:

"“The uçá (U. cordatus) lives hidden in the mud. The swimming crabs can also be found in burrows. The “aratu” sometimes gets in, but they are often found in stock hollows. Those old mangrove woods that are hollow, they go there, and go inside them” (R., 29 years old)."

"“The difference is that the “gaiamum” crab survives in the dry places, it lives in the bushes. It also uses water, right? Because no one can live without water, but it moves about the dry too” (R., 29 years old)."

The identification of ecological zones in fishing communities is a fairly common practice [22,37,65,67-69]. In a survey on the bio-ecological aspects of mangrove brachyuran in Itacorubi (Florianópolis-SC), Branco [70] has recorded a greater abundance of swimming crabs in the mouths of rivers that flow through the mangrove swamps, canals and streams, with peaks in spring and summer, while *G. cruentata* was widely found in the soil of intertidal zones and edges of rivers and canals where it builds burrows along the mangrove roots (peaks in summer); on the other hand *Cardisoma guanhumi* were more frequently observed in “apicum” (“salty water marshes”), where they build burrows above the high tide mark, with predominance in spring; finally, *Ucides cordatus* was more abundant in intertidal and infratidal zones, where they preferred to dig burrows due to lower variation in water salinity [71], also predominantly in spring. A subsequent study made by Branco [53], in the same mangrove, has recorded variations ranging from 90 to 180 cm, with an average depth of 120 cm in the burrows built by *U. cordatus*, depending on the area and the season; also, a higher average number of burrows per square meter was observed in the sandy bottom shoreline, which was directly related to fluctuations in water salinity in the burrows.

## Conclusion

Artisanal fishermen of Conde have an established knowledge on biological and ecological aspects related to brachyuran crustaceans of economic importance in the region, as well as the environment where they live, which covers the external morphology, life cycle associated with tidal dynamics, reproduction, trophic ecology and spatial and temporal distribution. Much of this knowledge is supported by data from the specialized scientific literature.

Although the creation of the Marine Extractive Reserve, a sort of marine protected area, IUCN category VI, to preserve both scenarios environmental and socio-cultural region of Earl is still a future possibility, this study, and other ethnobiological to be performed, should be used for ordering the management of local resources. In the case of the use of the crustaceans by local communities, the establishment of rules of use to the zones identified – “mangrove”, “river” and “marsh” should be incorporated into the protected area management, ensuring the sustainability of the resource and recognizing traditional knowledge.

With this interaction ensures the perpetuation of social local way of life and biodiversity conservation on the north coast of Bahia.

## Competing interests

The authors declare that they have no competing interests.

## Authors’ contributions

HFM carried out the field research and drafted the manuscript. EMCN participated in its design and coordination, and helped to draft the manuscript. AS helped to draft the manuscript. All authors read and approved the final manuscript.

## References

[B1] Cintrón G Schaeffer-Novelli Y Introducción a la ecología del manglar Montevideo 1983 UNESCO/ROSTLAC,

[B2] Oliveira LAK Freitas RR Barroso GF Manguezais: turismo e sustentabilidade Cadernos Virtual de Turismo 2005 5 51 56

[B3] FAO Forest Resources Assessment Working Paper No. 63. Forest Resources Division 2003 Wilkie, M.L. and Fortuna, S, FAO, Rome

[B4] Wielgus J Cooper E Torres R Burke L Coastal Capital: Dominican Republic. Case studies on the economic value of coastal ecosystems in the Dominican Republic. Working Paper 2010 World Resources Institute, Washington, DC http://www.wri.org Available online atcoastal-capital

[B5] Alves RRN Nishida AK A ecdise do caranguejo-uçá, Ucides cordatus L. (Decapoda, Brachyura) na visão dos caranguejeiros Interciencia 2002 27 110 117

[B6] Aveline LC Fauna dos manguezais brasileiros Revista Brasileira de Geografia 1980 42 786 821

[B7] Ruppert EE Fox S Barnes RD Zoologia dos invertebrados 2005 uma abordagem funcional-evolutiva São Paulo, Roca

[B8] Schaeffer-Novelli Y Manguezal 1995 ecossistema entre a terra e o mar São Paulo: Caribbean Ecological Research, São Paulo

[B9] Jankowsky M Pires JSR Nordi N Contribuição ao manejo participativo do caranguejo-uçá, Ucides cordatus (L., 1763), em Cananéia, SP Boletim do Instituto de Pesca 2006 32 221 228

[B10] Nascimento DM Mourão JS Rocha PD Ferreira EM Bezerra DM Impactos sócio-ambientais provocados pela técnica “redinha” na captura do caranguejo-uçá Ucides cordatus no estuário do Rio Mamanguape (PB) 2008 Resumos do IX Encontro de Biologia da UEFS; IV Encontro Nordestino de Etnoecologia e Etnobiologia, Feira de Santana: Universidade Estadual de Feira de Santana 34

[B11] Cascudo LC 1972 Dicionário do folclore brasileiro Rio de Janeiro, Ediouro

[B12] Farias MF Rocha-Barreira CA 2007 Conchas de moluscos no artesanato cearense Fortaleza, Nave

[B13] Reitermajer D Comunidade extrativista do manguezal de Porto Sauípe, Entre Rios-BA: uma abordagem ecológica e social 1996 Monografia Universidade Federal da Bahia, Salvador

[B14] Nomura H 2001 Os crustáceos na cultura popular Mossoró, Fundação Vingt-Un Rosado

[B15] Souto FJB A ciência que veio da lama 2004 Uma abordagem etnoecológica abrangente das relações ser humano/manguezal na comunidade pesqueira de Acupe, Santo Amaro-BA, Tese Universidade Federal de São Carlos, São Carlos

[B16] Lages-Filho J A medicina popular em Alagoas Salvador 1934 A medicina popular em Alagoas Salvador, Instituto Nina Rodrigues

[B17] Magalhães J Medicina folclórica Fortaleza 1966 Medicina folclórica Fortaleza, Imprensa Universitária do Ceará

[B18] Costa-Neto EM Gordiano-Lima KL Contribuição ao estudo da interação entre pescadores e caranguejos (Crustacea, Decapoda, Brachyura): considerações etnobiológicas em uma comunidade pesqueira do Estado da Bahia, Brasil Actualidades Biológicas 2000 22 195 202

[B19] Posey DA Etnobiologia: teoria e prática 1986 Petrópolis: Vozes. Ribeiro D, Brasileira 15 25

[B20] Costa-Neto EM A cultura pesqueira do litoral norte da Bahia 2001 etnoictiologia, desenvolvimento e sustentabilidade Salvador: EDUFBA,

[B21] Costa-Neto EM Etnoictiologia, desenvolvimento e sustentabilidade no litoral norte baiano 1998 Um estudo de caso entre pescadores do município de Conde. Msc, Thesis Universidade Federal de Alagoas, Maceió

[B22] Souto FJB Uma abordagem etnoecológica da pesca do caranguejo, Ucides cordatus, Linnaeus, 1763 (Decapoda: Brachyura), no manguezal do distrito de Acupe (Santo Amaro – BA) Biotemas 2007 20 69 80

[B23] Saraiva RS Aspectos etnoecológicos da pesca do pitu, Macrobrachium carcinus, Linnaeus, 1758 (Decapoda; Palaemonidae), no Rio Pojuca (Distrito de Barra do Pojuca, Camaçari – BA) 2008 Monografia (Licenciatura em Ciências Biológicas), Universidade Católica do Salvador, Salvador

[B24] Magalhães HF Etnoecologia de crustáceos (Decapoda: Brachyura) segundo os pescadores artesanais do município de Conde, litoral norte do Estado da Bahia 2009 Msc, Thesis, Universidade Estadual de Santa Cruz, Ilhéus

[B25] Costa-Neto EM Andrade CTS Couto DF Magalhães HF Mascarenhas LS Campos EVM Diagnóstico etnoecológico em comunidades pesqueiras do município de Conde, região Litoral Norte do Estado da Bahia 2010 Costa-Neto EM, Santos FM, Londero JC, Santa Cruz do Sul 20 52

[B26] Sturtevant WC Studies in ethnoscience American Anthropologist 1964 66 99 131

[B27] Goodman LA Amostragem bola de neve Annals of Mathematics and Statistics 1961 32 148 170 10.1214/aoms/1177705148

[B28] Fiscarelli AG Pinheiro MAA Perfil sócio-econômico e conhecimento etnobiológico do catador de caranguejo uçá, Ucides cordatus (Linnaeus, 1763) nos manguezais de Iguape (24º 41’ S), SP, Brasil Actualidades Biológicas 2002 24 129 142

[B29] Narchi W Crustáceos 1973 Polígono, estudos práticos São Paulo

[B30] Spradley JP McCurdy DW The cultural experience 1972 ethnography in complex society Tennessee, Kingsport Press of Kingsport

[B31] Marques JGW Aspectos ecológicos na etnoictiologia dos pescadores do Complexo Estuarino-Lagunar Mundaú-Manguaba 1991 Alagoas. Thesis Universidade Estadual de Campinas, Campinas, Campinas

[B32] Souza GS Tratado descritivo do Brasil em 2000 Recife, Massangana 1587

[B33] Brusca RC Brusca GJ Invertebrados Rio de Janeiro 2007 Invertebrados Rio de Janeiro, Guanabara Koogan

[B34] Pinheiro MAA Fiscarelli AG Manual de apoio à fiscalização do caranguejo-uçá (Ucides cordatus) Itajaí 2001 CEPSUL/IBAMA,

[B35] Pinto-Coelho RM Fundamentos em ecologia Porto Alegre 2000 Fundamentos em ecologia Porto Alegre, Artes Médicas Sul

[B36] Hickman CP Roberts LS Larson A Integrated principles of zoology New York 2001 McGraw-Hill, New York

[B37] Nordi N A captura do caranguejo-uçá (Ucides cordatus) durante o evento reprodutivo da espécie: o ponto de vista dos caranguejeiros Revista Nordestina de Biologia 1994 9 41 47

[B38] Alcântara-Filho P Contribuição ao estudo da biologia e ecologia do caranguejo-uçá, Ucides cordatus (L., 1763) (Crustacea, Decapoda, Brachyura), no manguezal do Rio Ceará (Brasil) Arquivos de Ciências Marinhas 1978 18 1 41

[B39] Nascimento AS 1993 Biologia do caranguejo-uçá Ucides cordatus Aracaju, ADEMA

[B40] Maneschy MC Pescadores nos manguezais: estratégias técnicas e relações sociais de produção na captura de caranguejo 1993 MCT/CNPq Furtado LG, Leitão W, Fiúza A, Amazônia Belém 19 62

[B41] Góes JM Fernandes-Góes LC Legat JFA Andada” do caranguejo Ucides cordatus (Linnaeus, 1763) (Crustacea, Ocypodidae) na Área de Proteção Ambiental (APA) do Delta do Parnaíba, Piauí 2005 Resumos do 15º Encontro de Zoologia do Nordeste, Salvador 217

[B42] Nordi N A produção dos catadores de caranguejo-uçá (Ucides cordatus) na região de Várzea Nova, Paraíba, Brasil Revista Nordestina de Biologia 1994 9 71 77

[B43] Sastry AN Ecological aspects of reproduction 1983 Press Bliss DE, Vernberg FJ, Vernberg WB, New York 179 270

[B44] Hartnoll RG Gould P Brachyuran life history strategies and the optimization of egg production Symposium of the Zoological Society of London 1988 59 1 9

[B45] Costa RS Biologia do caranguejo-uçá, Ucides cordatus (Linnaeus, 1763) – Crustáceo, decápode – no Nordeste brasileiro Boletim Cearense de Agronomia 1979 20 1 74

[B46] Mota-Alves MI Sobre a reprodução do caranguejo-uçá, Ucides cordatus (Linnaeus), em mangues do Estado do Ceará (Brasil) Arquivos de Ciências Marinhas 1975 15 85 91

[B47] Castro ACL Aspectos bio-ecológicos do caranguejo-uçá, Ucides cordatus cordatus (Linnaeus 1763), no estuário do rio dos Cachorros e estreito do Coqueiro, São Luís – MA Boletim do Laboratório de Hidrobiologia 1986 7 7 27

[B48] Dalabona G Loyola-Silva J Período reprodutivo de Ucides cordatus (Linnaeus) (Brachyura, Ocypodidae) na Baía das Laranjeiras, sul do Brasil Acta Bioloógica Paranaense 2005 34 115 126

[B49] Marques JGW Pescando pescadores 2001 ciência e etnociência em uma perspectiva ecológica São Paulo, NUPAUB/Fundação Ford

[B50] Paiva MP Bezerra RCF Fonteles-Filho AA Tentativa de avaliação dos recursos pesqueiros do Nordeste brasileiro Arquivos de Ciências Marinhas 1971 11 1 43

[B51] Leitão SN Schwamborn R Interações tróficas no canal de Santa Cruz 2000 Barros HM, Eskinazi-Leça E, Macedo SJ, Recife 163 180

[B52] Nordhaus I Wolff M Diele K Litter processing and population food intake of the mangrove crab Ucides cordatus in a high intertidal forest in northern Brazil Estuarine and Coastal Shelf Science 2006 67 239 250 10.1016/j.ecss.2005.11.022

[B53] Branco JO Aspectos bioecológicos do caranguejo Ucides cordatus (Linnaeus, 1763) (Crustacea, Decapoda) do manguezal do Itacorubi, Santa Catarina, BR Arquivos de Biologia e Tecnologia 1993 36 133 148

[B54] Branco JO Verani JR Dinâmica da alimentação natural de Callinectes danae Smith (Decapoda, Portunidae) na Lagoa da Conceição, Florianópolis, Santa Catarina, Brasil Revista Brasileira de Zoologia 1997 14 1003 1018 10.1590/S0101-81751997000400014

[B55] Petti MAV Papel dos crustáceos braquiúros na rede trófica da plataforma interna de Ubatuba, São Paulo (Brasil) Neritica 1997 11 123 137

[B56] Carqueija CRG Gouvêa EP Hábito alimentar de Callinectes larvatus Ordway (Crustacea, Decapoda, Portunidae) no manguezal de Jiribatuba, Baía de Todos os Santos, Bahia Revista Brasileira de Zoologia 1998 15 273 278 10.1590/S0101-81751998000100023

[B57] Gonçalves P Rosa EA Minnie KY Estimativa populacional do caranguejo estuarinoGoniopsis cruentatano manguezal de Ratones, Florianópolis, SC 2007 Semana de Ensino, Pesquisa e Extensão da UFSC, Florianópolis

[B58] Branco JO Lunardon-Branco MJ Ecologia trófica de Portunus spinimanus Latreille, 1819, na Armação do Itacoporoy, Penha, Santa Catarina Revista Brasileira de Zoologia 2002 19 723 729 10.1590/S0101-81752002000300009

[B59] Gaspar MH Contribuição ao estudo biológico do “siri” Callinectes danae Smith, 1869 (Decapoda: Portunidae) do rio Itiberê (Paranaguá-PR) 1981 Msc. Thesis, Universidade Federal do Paraná, Curitiba

[B60] Nogueira-Ferreira FH Augusto SC Amplitude de nicho e similaridade no uso de recursos florais por abelhas eussociais em uma área de cerrado Bioscience Journal 2007 23 45 51

[B61] Pinheiro MMA Fransozo A Negreiros-Fransozo ML Dimensionamento e sobreposição de nichos dos portunídeos (Decapoda, Brachyura), na Enseada da Fortaleza, Ubatuba, São Paulo, Brasil Revista Brasileira de Zoologia 1997 14 371 378 10.1590/S0101-81751997000200010

[B62] Nomura H Os mamíferos no folclore Mossoró 1996 Fundação Vingt-Un Rosado, Fundação

[B63] Emmons LH Feers F Neotropical rainforest mammals 1990 The University of Chicago Press, A field guide Chicago

[B64] Carvalho JO Barbosa C Tosatti M Caracterização da dieta alimentar da Lontra longicaudis em um ambiente marinho, Praia de Naufragados, Ilha de Santa Catarina, SC-Brasil 2005 Resumos do IV Congresso Integrado de Iniciação Científica, Rio do Sul 32

[B65] Mourão JS Nordi N Pescadores, peixes, espaço e tempo: uma abordagem etnoecológica Interciencia 2006 31 358 363

[B66] Posey DA Etnobiologia e ciência de folk: sua importância para a Amazônia Tübinger Geographische Study 1987 95 95 108

[B67] Cordell J The lunar-tide fishing cycle in Northeastern Brazil Ethnology 1974 13 379 392 10.2307/3773053

[B68] Robben ACGM Sea tenure and conservation of coral reef resources in Brazil Cultural Survival Quarterly 1985 9 45 47

[B69] Mourão JS Classificação e ecologia de peixes estuarinos por pescadores do estuário do rio Mamanguape – PB 2000 PhD Thesis Universidade Federal de São Carlos, São Carlos

[B70] Branco JO Aspectos ecológicos dos Brachyura (Crustacea: Decapoda) no manguezal do Itacorubi, SC, Brasil Revista Brasileira de Zoologia 1991 7 165 179

[B71] Soriano-Sierra EJ Macedo-Silva JRB Derner RB Branco JO Ecologia e Gerenciamento do Manguezal de Itacorubí Florianópolis: NEMAR/CCB/UFSC, SDM/FEPEMA 1998 Soriano-Sierra EJ, Sierra de Ledo B, Aspectos ecológicos do Manguezal de Itacorubí, Santa Catarina, Brasil 115 138

